# Acute post-exercise change in blood pressure and exercise training response in patients with coronary artery disease

**DOI:** 10.3389/fphys.2014.00526

**Published:** 2015-01-12

**Authors:** Antti M. Kiviniemi, Arto J. Hautala, Jaana J. Karjalainen, Olli-Pekka Piira, Samuli Lepojärvi, Olavi Ukkola, Heikki V. Huikuri, Mikko P. Tulppo

**Affiliations:** ^1^Department of Exercise and Medical Physiology, Verve ResearchOulu, Finland; ^2^Medical Research Center, University of Oulu, Oulu University HospitalOulu, Finland; ^3^Department of Applied Sciences, London South Bank UniversityLondon, UK

**Keywords:** ischemic heart disease, hypertension, arterial pressure, training adaptation, acute exercise, exercise testing, autonomic function

## Abstract

We tested the hypothesis that acute post-exercise change in blood pressure (BP) may predict exercise training responses in BP in patients with coronary artery disease (CAD). Patients with CAD (*n* = 116, age 62 ± 5 years, 85 men) underwent BP assessments at rest and during 10-min recovery following a symptom-limited exercise test before and after the 6-month training intervention (one strength and 3-4 aerobic moderate-intensity exercises weekly). Post-exercise change in systolic BP (SBP) was calculated by subtracting resting SBP from lowest post-exercise SBP. The training-induced change in resting SBP was −2 ± 13 mmHg (*p* = 0.064), ranging from −42 to 35 mmHg. Larger post-exercise decrease in SBP and baseline resting SBP predicted a larger training-induced decrement in SBP (β = 0.46 and β = −0.44, respectively, *p* < 0.001 for both). Acute post-exercise decrease in SBP provided additive value to baseline resting SBP in the prediction of training-induced change in resting SBP (R^2^ from 0.20 to 0.26, *p* = 0.002). After further adjustments for other potential confounders (sex, age, baseline body mass index, realized training load), post-exercise decrease in SBP still predicted the training response in resting SBP (β = 0.26, *p* = 0.015). Acute post-exercise change in SBP was associated with training-induced change in resting SBP in patients with CAD, providing significant predictive information beyond baseline resting SBP.

## Introduction

Elevated BP is an important therapeutic target in cardiovascular risk management in coronary artery disease (CAD) (Yap et al., 2007). In addition to medical treatment, lifestyle interventions, including exercise training, are of pivotal importance in treatment of increased BP (Eckel et al., 2014). Exercise-based rehabilitation decreases mortality and resting BP by an average of ~3 mmHg in patients with CAD, but many patients receive no benefits to BP from training (Hagberg et al., 2000; Rice et al., 2002; Taylor et al., 2004), which limits the effectiveness of exercise training in prevention and treatment of hypertension in CAD.

Rationale for the effects of exercise training on BP relies partly on acute BP reduction after a single exercise—often termed post-exercise hypotension—which typically constitutes BP decreases of 5–20 mmHg for hours after the exercise (Macdonald et al., 2000; Halliwill, 2001; Jones et al., 2007; Eicher et al., 2010). Strong relationship has been observed between acute post-exercise hypotension and chronic reductions in BP among pre-hypertensive individuals, suggesting that acute post-exercise hypotension might well identify achievable decrease in BP by exercise training (Liu et al., 2012). However, Liu et al. did not report to what extent acute post-exercise hypotension predicts training-induced decrements in BP beyond baseline BP (Liu et al., 2012), which often determines both acute and chronic effects of exercise on BP (Taylor et al., 2010). It is not known whether acute post-exercise change in BP is related to training outcome in resting BP among patients with CAD when baseline resting BP is considered. We hypothesized that acute post-exercise change in BP may be related to training-induced changes in resting BP in CAD, providing significant information beyond baseline resting BP.

## Materials and methods

### Subjects and study protocol

The present study is part of the ARTEMIS study (Innovation to Reduce Cardiovascular Complications of Diabetes at the Intersection) in Division of Cardiology at Oulu University Hospital (Oulu, Finland) and Department of Exercise and Medical Physiology at Verve (Oulu, Finland). The ARTEMIS study is registered at ClinicalTrials.gov, Record 1539/31/06. The study was performed according to the Declaration of Helsinki, the local research ethics committee of the Northern Ostrobothnia Hospital District approved the protocol, and all the subjects gave their written informed consent.

Patients with angiographically documented CAD (*n* = 146) and willing to participate exercise training intervention were recruited from the ARTEMIS database, observing the exclusion criteria described elsewhere in details (Kiviniemi et al., 2013). Patients with pre-diabetes, verified by fasting glucose and oral glucose tolerance test (Alberti and Zimmet, 1998), were excluded because of the main focus of the ARTEMIS project. CAD and its severity were assessed by syntax score (Sianos et al., 2005). Left ventricular mass index and systolic (ejection fraction) and diastolic function (ratio of early transmitral flow velocity to early diastolic mitral annulus velocity) were measured with two-dimensional tissue Doppler echocardiography (Vivid 7, GE Healthcare, Wauwatosa, WI, USA). Urine and fasting blood samples were obtained for analysis of renal function, inflammation, lipid and glucose metabolism. These clinical examinations were conducted only at the baseline.

The patients were invited to the Department of Exercise and Medical Physiology at Verve (Oulu, Finland), where further measurements were conducted before and after the intervention. The tests were performed at the same time of day in pre- and post-training conditions when subjects were refrained from eating and caffeine for 3 h and from exercise and alcohol for 24 h before the tests but did not cease their medications. The patients underwent measurements at supine rest and during passive head-up tilt to determine resting BP and cardiac autonomic function. After 10–15 min of supine rest, the tilt protocol started with supine recording (5 min) followed by passive tilt (80°, 5 min), while the patients breathing spontaneously. Blood pressure was measured twice during both phases (at 1:30 and 4:00) of the protocol (Tango, SunTech, Raleigh, NC, USA) and the average value of the two measurements was used to represent BP for each phase, with BP in the supine position representing resting BP. R-R intervals were recorded at an accuracy of 1 ms (Polar R-R recorder, Polar Electro Oy, Kempele, Finland).

The patients performed a symptom-limited maximal exercise test on a bicycle (Monark Ergomedic 839 E, Monark Exercise Ab, Vansbro, Sweden) for assessment of cardiorespiratory fitness and BP during the exercise and recovery. The test was started at 30 W and the work rate was increased by 15 W in men and 10 W in women every minute until voluntary exhaustion or ST segment depression >0.2 mV on ECG (GE Healthcare, CAM-14, Freiburg, Germany). After termination of the test, the patients lay down and remained still and silent for 10 min. Blood pressure was measured at 2-min intervals during the test and recovery, where the first BP reading was obtained 1 min after exhaustion (Tango, SunTech, Raleigh, NC, USA). The highest 1-min mean value of oxygen consumption (M909 Ergospirometer, Medikro, Kuopio, Finland) was taken to express peak oxygen uptake (VO_2peak_). Exercise capacity was calculated in metabolic equivalents (METs) from the mean workload during the last minute of the test. Relative exercise capacity was expressed as a percentage of the predicted exercise capacity, which was calculated as 18–(0.15·age) for men and 14.7–(0.13·age) for women (Kim et al., 2007). Acute post-exercise change in BP was quantified by subtracting the mean of two resting BP measurements measured before the exercise test from the lowest BP value during 10-min supine recovery after symptom-limited exercise both measured in a supine position (ΔBP_PostEx_ = resting BP–minimum post-exercise BP).

### Heart rate variability analysis

Heart rate variability was edited and analyzed in Hearts program (Heart Signal Co., Oulu, Finland). Ectopic beats and artifacts were removed from the tachogram based on visual inspection (<10% for each recording). In addition to mean heart rate (HR), autoregressive spectral analysis (model order 20) was performed to analyze the low- (LF, 0.04–0.15 Hz) and high-frequency (HF, 0.15–0.4 Hz) power of R-R interval oscillation from both entire 5-min phases (Taskforce, 1996). The power spectrum densities are presented in absolute units transformed into natural logarithm (ln ms^2^). The LF/HF ratio was also calculated.

### Training intervention

Training during the first 3 months included three HR-controlled (Polar F1, Polar Electro Oy, Kempele, Finland) endurance exercises (30 min, 50–60% of the HR reserve) and one strength exercise (30 min) weekly. The patients were allowed to choose the mode of endurance exercise which was typically walking, jogging or cycling. Strength exercise was circuit training including major muscle groups at moderate intensity (2*7 sets, ≥10 repetitions/set). After 3 months, the training included two endurance exercises at 50–60% and two at 60–70% of the HR reserve (both 30 min), and one strength training session (30 min). The patients received a diary with prescribed training days, along with duration and intensity (HR) of the exercises, and resting days and marked the realized training mode, duration, and mean HR in the diary. The patients were contacted after 1 and 3 months by a specialist in sports medicine to check their progress. Weekly target and realized training loads were calculated as the mean training impulse (TRIMP) (Morton et al., 1990). In calculating the target TRIMP for endurance training, the intensity was an average of prescribed exercise HR (55 or 65% of the HR reserve) and in strength training, on 90% of the target HR of light endurance exercise, based on our previous experience (Hautala et al., 2006). Thirty patients terminated the study due to personal reasons, a lack of motivation, or musculoskeletal problems, thus, the final analysis consisted of 116 patients (Table 1).

**Table 1:** **Characteristics of the study group**.

	***n* = 116**
Men, n	87 (75%)
Age, years	62 ± 5
Hypertension, n	81 (70%)
Current smokers, n	9 (8%)
CCS class ≥ 2, n	18 (16%)
T2D, n	53 (46%)
T2D duration, months	70 ± 84
**HISTORY OF AMI**	
NSTEMI, n	31 (27%)
STEMI n	22 (19%)
**REVASCULARIZATION**	
PCI, n	74 (64%)
CABG, n	26 (22%)
**ECHOCARDIOGRAPHY**	
LVEF, %	66 ± 8
LVMI	98 ± 22
E/E′	9.5 ± 3.0
**CORONARY ANGIOGRAPHY**	
Syntax score	3.6 ± 5.0
**MEDICATION**	
Oral antidiabetics, n	41 (35%)
Insulin, n	5 (4%)
Beta blockers, n	103 (89%)
ACEI or ARB, n	67 (58%)
Anticholesterol agents, n	107 (92%)
Anticoagulants, n	113 (97%)
Calcium antagonists, n	19 (16%)
Nitrates, n	27 (23%)
Diuretics, n	37 (32%)
**LABORATORY ANALYSES**	
Fasting glucose, mmol·L^−1^	6.0 ± 1.1
HbA1c, %	6.2 ± 0.7
Insulin, mU·L^−1^	14.0 ± 10.2
Total cholesterol, mmol·L^−1^	4.0 ± 0.8
LDL cholesterol, mmol·L^−1^	2.4 ± 0.7
HDL cholesterol, mmol·L^−1^	1.2 ± 0.3
Triglycerides, mmol·L^−1^	1.5 ± 0.8
hs-CRP, mg·L^−1^	1.9 ± 3.0
Creatinine, μmol·L^−1^	74 ± 14
Albumin, mg·L^−1^	10.4 ± 20.7
ACR, mg·mmol^−1^	1.2 ± 2.3

*Values are the number of subjects (proportion) or means ± SD. CCS, Canadian Cardiovascular Society angina classification; T2D, type 2 diabetes; AMI, acute myocardial infarction; NSTEMI, non-ST segment elevation myocardial infarction; STEMI, ST segment elevation myocardial infarction; PCI, percutaneus coronary intervention; CABG, coronary artery by-pass grafting; LVEF, left ventricular ejection fraction; LVMI, left ventricular mass index; E/E′, ratio of early transmitral flow velocity to early diastolic mitral annulus velocity, ACEI, angiotensin conversion enzymes inhibitor; ARB, angiotensin receptor blocker; HbA1c, glycated hemoglobin; LDL, low-density lipoprotein; HDL, high-density lipoprotein; hs-CRP, high-sensitivity C-reactive protein; ACR, albumin-creatinine ratio*.

### Statistical analysis

The data are presented as the mean ± SD. The changes in antihypertensive drug treatment during the intervention were coded as follows: −2 = terminated, −1 = decreased dose, 0 = no change, 1 = increased dose, and 2 = start of new medication. The paired t-test was used to assess the effects of training on measured variables, except for time to minimum post-exercise BP which was tested by Wilcoxon test due to its non-Gaussian distribution. Univariate linear regression was employed to determine the relationship between the change in resting BP and baseline variables and their changes during the intervention as well as to assess the determinants of ΔBP_PostEx_ at the baseline. Multivariate linear regression was used to assess the additive value of ΔBP_PostEx_ to baseline resting BP in the prediction of training response in resting BP (model 1). The most significant determinants of training responses in resting BP were analyzed by multivariate linear regression, where baseline resting BP, baseline ΔBP_PostEx_, age, sex, baseline body mass index (BMI), realized training load, and other relevant variables and their changes, specifically associated with training responses in SBP and DBP, were entered as independent factors (model 2). Multivariate linear regression was also employed to assess the most significant determinants of ΔBP_PostEx_ at the baseline by entering the significant variables in univariate analysis as independent factors. The data were analyzed using IBM SPSS Statistics 21 (IBM Corporation, Somers, New York). A *p* < 0.05 was considered statistically significant.

## Results

The mean TRIMP was 262 ± 155/week, which was 129 ± 74% of the target TRIMP (median: 110%; interquartile range: 85–161%). Training frequencies and durations were 3.4 ± 1.1, 2.8 ± 1.0 and 0.7 ± 0.4 sessions/week; and 38 ± 15, 47 ± 16, and 27 ± 13 min for all exercises, endurance and strength exercises, respectively. The most significant changes were observed in BMI, waist-hip ratio, resting DBP, exercise capacity, and VO_2peak_ (Table 2). A reduction of ≥2 mmHg in SBP was observed in 46% of the patients, whereas 59% of the patients obtained DBP reduction of ≥1 mmHg during the intervention. No significant changes were observed in antihypertensive medication.

**Table 2:** **Effects of exercise training on anthropometric, blood pressure, heart rate variability, and exercise test variables**.

	**Pre**	**Post**	***p*-Value**
Body mass index, kg·m^−2^	28.2 ± 3.9	27.9 ± 3.8	0.003
Waist-hip ratio	0.97 ± 0.07	0.96 ± 0.07	0.001
**SUPINE REST**			
SBP, mmHg	137 ± 17	134 ± 16	0.064
DBP, mmHg	82 ± 8	80 ± 7	0.024
HR, bpm	56 ± 8	56 ± 8	0.269
LF, ln ms^2^	5.6 ± 1.2	5.5 ± 1.1	0.356
HF, ln ms^2^	5.3 ± 1.2	5.3 ± 1.1	0.789
LF/HF-ratio	1.9 ± 1.9	1.8 ± 2.2	0.767
**PASSIVE TILT**			
SBP, mmHg	127 ± 20	126 ± 18	0.333
DBP, mmHg	77 ± 10	77 ± 10	0.277
HR, bpm	61 ± 8	61 ± 9	0.732
LF, ln ms^2^	5.4 ± 1.1	5.3 ± 1.1	0.801
HF, ln ms^2^	4.9 ± 1.2	5.0 ± 1.3	0.444
LF/HF-ratio	2.2 ± 1.6	2.2 ± 2.1	0.927
**EXERCISE TEST**			
EC, METs	7.4 ± 1.9	7.6 ± 2.0	<0.001
EC, %	90 ± 22	93 ± 22	<0.001
VO_2peak_, L·min^−1^	2.00 ± 0.55	2.06 ± 0.57	<0.001
VO_2peak_, mL·kg^−1^·min^−1^	24.6 ± 6.7	25.6 ± 7.0	<0.001
HR_peak_, bpm	134 ± 19	135 ± 19	0.502
SBP_peak_, mmHg	209 ± 28	208 ± 29	0.671
DBP_peak_, mmHg	99 ± 13	99 ± 12	0.638
ΔSBP_PostEx_, mmHg	−13 ± 14	−12 ± 14	0.238
ΔDBP_PostEx_, mmHg	−11 ± 7	−10 ± 7	0.263
Time_PostExSBP_, min	8 ± 1	8 ± 1	0.646
Time_PostExDBP_, min	7 ± 2	6 ± 3	0.359

*Values are or means ± SD. SBP, systolic blood pressure; DBP, diastolic blood pressure; HR, heart rate; LF, low frequency power of heart rate variability; HF, high frequency power of heart rate variability; EC, exercise capacity; VO_2peak_, peak oxygen consumption; PostEx, post-exercise*.

In addition to baseline resting SBP, ΔSBP_PostEx_ was strongly related to training response in SBP (Table 3). The training-induced change in resting DBP was associated with baseline resting DBP, ΔDBP_PostEx_, syntax score, nitrate medication, albumin, albumin-creatinine ratio, LF/HF ratio, more strongly during passive tilt (Table 3), and with the change in relative VO_2peak_ (β = −0.20, *p* = 0.036). The changes in BMI, antihypertensive medication, and heart rate variability were not associated with training outcome in SBP or DBP.

**Table 3:** **Univariate determinants of acute post-exercise change in blood pressure and training-induced change in resting blood pressure at the baseline**.

	**ΔSBP_PostEx_**	**ΔDBP_PostEx_**	**ΔSBP_Training_**	**ΔDBP_Training_**
Male gender	0.20^*^	0.07	0.17	−0.07
Age	−0.01	0.12	−0.18	−0.03
Body mass index	0.19^*^	0.16	0.08	−0.14
Waist-hip ratio	0.05	0.03	0.07	−0.14
Hypertension	−0.09	−0.05	−0.09	−0.06
Current smoking	−0.12	−0.06	0.10	−0.05
CCS class ≥ 2	0.01	0.04	0.13	−0.01
T2D	0.20^*^	0.20^*^	0.11	−0.04
History of myocardial infarction	0.08	0.20^*^	0.07	0.12
History of PCI or CABG	0.08	0.10	0.09	0.10
LVEF	0.00	−0.03	0.05	0.12
LVMI	0.06	0.07	0.09	−0.14
E/E′	−0.04	−0.07	0.04	−0.00
Syntax score	−0.01	−0.02	−0.08	−0.30^**^
**MEDICATION**				
Oral antidiabetics	0.17	0.19^*^	0.07	0.01
Insulin	−0.04	−0.10	0.13	0.02
Beta blockers	0.09	0.13	0.02	0.12
ACEI or ARB	0.01	−0.02	0.05	0.04
Anticholesterol agents	−0.04	0.05	−0.08	0.08
Anticoagulants	−0.17	−0.07	−0.09	−0.01
Calcium antagonists	0.06	0.06	0.06	0.06
Nitrates	0.15	0.02	0.12	−0.23^*^
Diuretics	−0.11	0.01	−0.01	0.05
**LABORATORY ANALYSES**				
Fasting glucose	0.15	0.12	0.06	−0.07
HbA1c	0.10	0.20^*^	−0.04	−0.02
Insulin	0.02	0.01	0.04	0.05
Total cholesterol	−0.17	−0.08	−0.06	−0.06
LDL cholesterol	−0.20^*^	−0.09	−0.02	−0.03
HDL cholesterol	−0.10	−0.12	0.00	0.06
Triglycerides	0.11	0.21^*^	−0.08	−0.07
hs-CRP	0.04	0.00	0.06	0.08
Creatinine	0.26	0.07	−0.08	−0.16
Albumin	−0.01	0.03	−0.13	−0.30^**^
ACR	−0.06	−0.02	−0.15	−0.31^**^
**SUPINE REST**				
SBP	−0.55^***^	−0.38^***^	−0.44^***^	−0.28^**^
DBP	−0.37^***^	−0.54^***^	−0.27^**^	−0.55^***^
HR	−0.09	−0.15	−0.10	−0.14
LF	−0.12	−0.09	0.01	−0.09
HF	−0.07	−0.08	0.09	−0.01
LF/HF-ratio	−0.09	−0.09	−0.10	−0.20^*^
**PASSIVE TILT**				
SBP	−0.38^***^	−0.32^**^	−0.20^*^	−0.17
DBP	−0.32^**^	−0.35^***^	−0.24^*^	−0.30^**^
HR	−0.04	−0.08	−0.07	−0.12
LF	−0.23^*^	−0.16	−0.06	−0.07
HF	−0.20^*^	−0.08	0.02	0.02
LF/HF-ratio	−0.06	−0.20^*^	−0.11	−0.24^**^
**EXERCISE TEST**				
EC, METs	0.07	−0.13	0.05	−0.08
EC, %	−0.03	−0.13	−0.08	−0.07
VO_2peak_, L·min^−1^	0.19^*^	−0.01	0.15	−0.12
VO_2peak_, mL·kg^−1^·min^−1^	0.08	−0.12	0.07	−0.08
HR_peak_	−0.10	−0.23^*^	−0.10	−0.08
SBP_peak_	−0.06	−0.17	−0.11	−0.13
DBP_peak_	−0.18	−0.10	−0.22^*^	−0.02
ΔSBP_PostEx_	−	0.56^***^	0.46^***^	0.21^*^
ΔDBP_PostEx_	0.56^***^	−	0.15	0.35^***^

*Values are standardized coefficients (β) from univariate linear regression. CCS, Canadian Cardiovascular Society angina classification; T2D, type 2 diabetes; PCI, percutaneus coronary intervention; CABG, coronary artery by-pass grafting; LVEF, left ventricular ejection fraction; LVMI, left ventricular mass index; E/E′, ratio of early transmitral flow velocity to early diastolic mitral annulus velocity; ACEI, angiotensin conversion enzymes inhibitor; ARB, angiotensin receptor blocker; HbA1c, glycated hemoglobin; LDL, low-density lipoprotein; HDL, high-density lipoprotein; hs-CRP, high-sensitivity C-reactive protein; ACR, albumin-creatinine ratio; SBP, systolic blood pressure; DBP, diastolic blood pressure; HR, heart rate; LF, low frequency power of heart rate variability; HF, high frequency power of heart rate variability; EC, exercise capacity; VO_*2peak*_, peak oxygen consumption; PostEx, post-exercise*.

* p < 0.05,

** p < 0.01,

*** *p < 0.001*.

**Figure 1:**
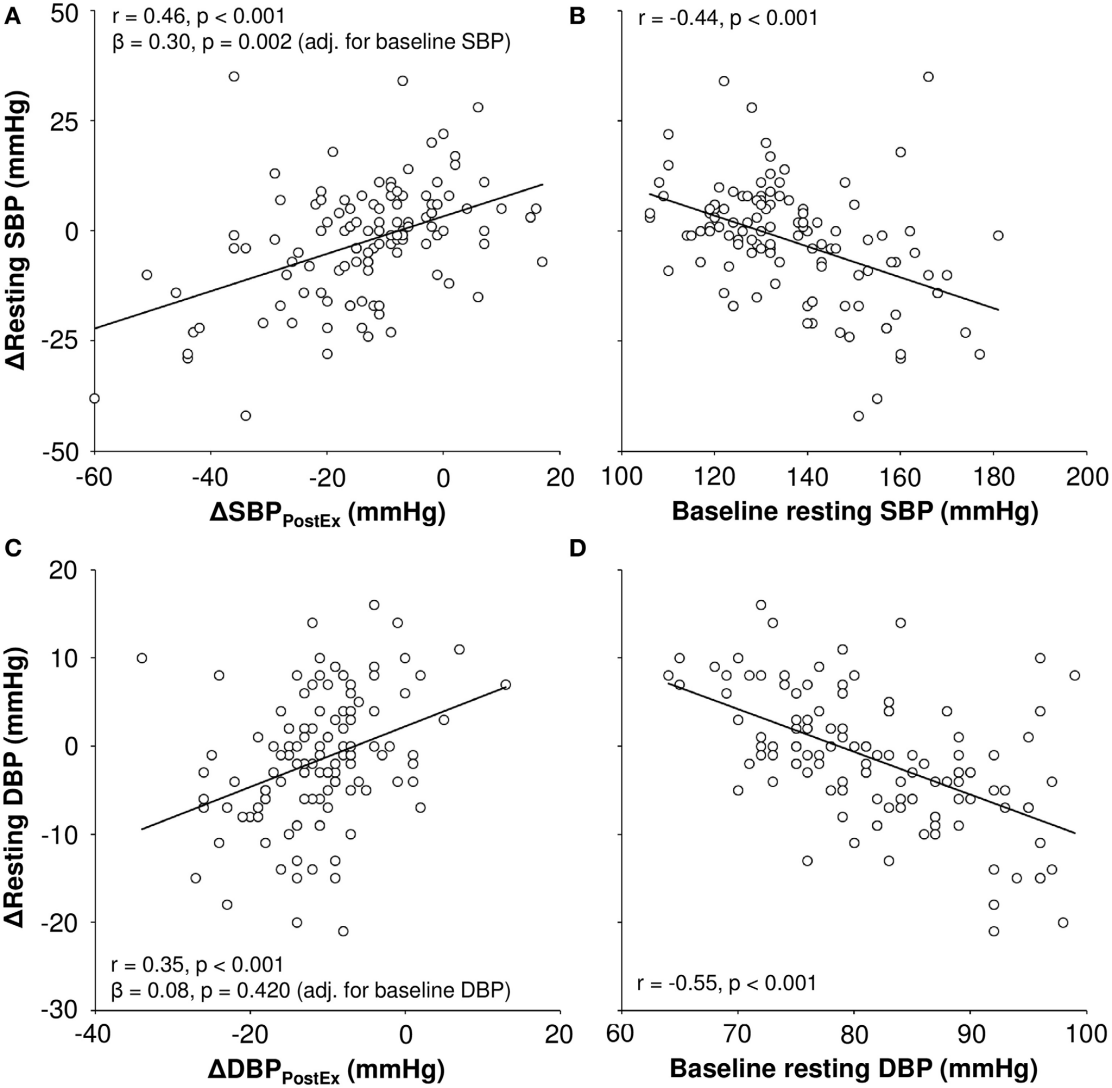
**Correlation of acute post-exercise change (ΔBP_PostEx_) in blood pressure and baseline resting blood pressure to exercise training-mediated changes in resting systolic (SBP, A,B) and diastolic blood pressure (DBP, C,D)**.

ΔSBP_PostEx_ provided significant additive value to baseline resting SBP in the prediction of training response in resting SBP (R^2^ from 0.20 to 0.26, *p* = 0.002, Figure 1). In multivariate linear regression, the final model explained 30% of training response in SBP. ΔSBP_PostEx_ (β = 0.26, *p* = 0.015) and baseline resting SBP (β = −0.26, *p* = 0.010) were significant predictors of training outcome in SBP, while sex, age, baseline BMI, and realized training load were not (*p* > 0.05 for all).

ΔDBP_PostEx_ did not improve the prediction of training response in resting DBP after adjustment for baseline resting DBP (R^2^ from 0.30 to 0.31, *p* = 0.420). In the final model of training response in DBP, baseline values of resting DBP (β = −0.33, *p* = 0.001) and LF/HF ratio during passive tilt (β = −0.17, *p* = 0.041) and the change in relative VO_2peak_ (β = −0.18, *p* = 0.028) remained as significant factors, explaining 47% of training response in resting DBP. Sex, age, baseline BMI, training load, severity of CAD (syntax score), nitrate medication, renal function, and ΔDBP_PostEx_ were not significantly associated with training outcome in DBP in the multivariate regression (*p* > 0.05 for all).

The determinants of ΔBP_PostEx_ at the baseline are presented in Table 3. In multivariate analysis, resting SBP (β = −0.54, *p* < 0.001), type 2 diabetes (β = 0.18, *p* = 0.041) and BMI (β = 0.19, *p* = 0.022) remained as significant factors underlying ΔSBP_PostEx_, whereas sex, low-density lipoprotein cholesterol and absolute VO_2peak_ did not (*p* > 0.05 for all). Resting DBP (β = −0.51, *p* < 0.001) and triglycerides (β = 0.17, *p* = 0.043) were significant determinants of ΔDBP_PostEx_ in multivariate analysis but type 2 diabetes, history of myocardial infarction, HR_peak_, oral antidiabetic medication and glycated hemoglobin were not (*p* > 0.05 for all).

## Discussion

The main finding of the present study was that acute post-exercise change in SBP predicts training outcome in resting SBP beyond basal resting SBP in CAD and other potent determinants of training response in resting SBP. In contrast, acute post-exercise change in DBP was not as strongly related to training response in resting DBP. Interestingly, training outcome in DBP was associated with the baseline LF/HF ratio and improved VO_2peak_. The present results suggest that acute post-exercise change in BP contributes to the inter-individual variation in SBP responses to aerobic-based exercise training in CAD but to a lesser extent to training responses in DBP.

As expected (Hagberg et al., 2000; Rice et al., 2002; Liu et al., 2012), exercise training responses in BP involved large inter-individual variation in patients with CAD. Our principal finding was that acute post-exercise change in SBP predicted training outcome in resting SBP beyond baseline resting SBP in CAD. After inclusion of other determinants of training response in SBP, acute post-exercise change in SBP still predicted the training response in resting SBP. The present findings support the rationale connecting the acute and chronic SBP-lowering effects of exercise training (Halliwill, 2001; Liu et al., 2012), which was not completely explained by baseline resting SBP. Baseline resting BP must be taken into account in order to minimize the effects of mathematical coupling between acute and chronic BP responses (Taylor et al., 2010). In this light, the previous study by Liu et al. (2012) may have provided overly optimistic results. The correlation between acute post-exercise change in BP and training-induced changes in BP was lower in the present patients with CAD (β = 0.46 for SBP) compared with the previous pre-hypertensive group (β = 0.89 for SBP) (Liu et al., 2012). Greater heterogeneity in the present study population may also explain this lower correlation. Also, Liu et al. assessed acute post-exercise change in BP after typical aerobic exercise (30 min at 65% of maximal oxygen consumption) (Liu et al., 2012), whereas, in the present study, it was assessed after symptom-limited exercise test. Yet, acute post-exercise change in SBP predicted training outcome in resting SBP after adjustment for baseline resting SBP, suggesting that the present findings could be easily implemented in standard clinical exercise testing. If lesser post-exercise hypotension is observed, more frequent follow-up might be needed in order to tailor exercise training in order to optimize training responses in BP. Different training modalities, including, e.g., larger component of dynamic or isometric resistance training, may prove beneficial in this respect (Cornelissen et al., 2011; Cornelissen and Smart, 2013).

While the acute post-exercise change in SBP predicted the training response in resting SBP, this association was weaker in DBP. It is well-established that arterial stiffness is a major determinant of elevated SBP with advancing age, by augmenting pulse pressure due to loss of elasticity of the arteries (Franklin et al., 1997). While the contribution of systemic vascular resistance to DBP might be larger than to SBP, increased arterial stiffness may actually decrease DBP with advancing age >50 years, despite increasing SBP (Franklin et al., 1997). Exercise training decreases arterial stiffness (Joyner, 2000; Tabara et al., 2007), which may be a potent mechanism coupling acute post-exercise change in SBP and training-induced outcome in SBP—especially when the relationship between acute and the chronic effects of exercise were dissociated between SBP and DBP. While Tabara et al. did not observe decreases in arterial stiffness after acute aerobic exercise, a larger acute decrease in arterial stiffness was associated with a larger decrease in arterial stiffness after 6 months of exercise training among elderly individuals (Tabara et al., 2007).

While training outcome in DBP was most strongly determined by baseline resting DBP, increased LF/HF ratio, an estimate of sympathovagal balance (Malliani et al., 1991), and the training-induced increase in VO_2peak_ were associated with decreased DBP with training. The finding on LF/HF ratio suggests that baseline sympathetic predominance may predict the larger training-induced decrease in DBP. While DBP may be more associated with systemic vascular resistance—where the neural sympathetic component plays an important role—it could be hypothesized that patients with sympathetic predominance may have greater potential to decrease neural sympathetic activity. While the changes in LF/HF ratio were not associated with training outcome in DBP, contribution of autonomic balance to BP responses remains unclear. Interestingly, an improved relative VO_2peak_ was also associated with larger decrements in DBP. However, the changes in absolute VO_2peak_ and exercise capacity were not related to training-induced decreases in DBP, which is why the present results are inconsistent in this respect.

In conclusion, acute post-exercise change in BP predicted training outcome in resting SBP and provided significant information beyond baseline resting SBP in patients with CAD. However, post-exercise hypotension in DBP was not as strongly related to training response in resting DBP, which was most evidently explained by baseline resting DBP. The present findings help to understand the determinants underlying the inter-individual variation in BP responses to aerobic-based exercise training in CAD.

### Limitations

Control group would have improved the present research setting. Despite the well-controlled BP measurements, it would have been beneficial to establish how reproducibility of BP measurements contributes to the present observations. A possible white-coat effect might have occurred in some cases in laboratory BP assessments (Grassi et al., 1999). Also, due the considerable daily variation in BP, ambulatory measurements might have been beneficial. The changes in the resting BP and cardiorespiratory fitness at group level were only modest despite the well-realized training load. However, the training frequency turned out to be lower than currently recommended for the patients with CAD (Piepoli et al., 2010), which may explain these modest responses. Medical treatment, e.g., statin medication (92% of patients), may also have compromised the training responses in cardiorespiratory fitness (Mikus et al., 2013). Acute post-exercise change in BP was evaluated from the symptom-limited exercise stress test. Ultimately, we cannot establish how typically the realized exercise elicited acute post-exercise changes in BP. However, it is plausible to assume that the intensity and total workload of the exercise was in proportion to the patients' exercise capacity. Also, the present results rely on a rather short period of acute recovery when establishing acute post-exercise change in BP. As measured and expected, however, significant post-exercise hypotension was observed during the 10-min recovery (Macdonald et al., 1999). Finally, measures of systemic vascular resistance, arterial stiffness, and endothelial function might have provided more mechanistic insight to the present observations.

## Author contributions

Antti M. Kiviniemi: Design of the work, acquisition, analysis and interpretation of data, drafting the work, final approval of work and its integrity. Arto J. Hautala: Design of the work, acquisition, analysis and interpretation of data, revision and final approval of work and its integrity. Jaana J. Karjalainen: Design of the work, acquisition, analysis and interpretation of data, revision and final approval of work and its integrity. Olli-Pekka Piira: Design of the work, acquisition and interpretation of data, revision and final approval of work and its integrity. Samuli Lepojärvi: Design of the work, acquisition and interpretation of data, revision and final approval of work and its integrity. Olavi Ukkola: Design of the work, interpretation of data, revision and final approval of work and its integrity. Heikki V. Huikuri: Design of the work, interpretation of data, revision and final approval of work and its integrity. Mikko P. Tulppo: Design of the work, interpretation of data interpretation of data, revision and final approval of work and its integrity.

## Grants

This study was supported by funding from the Finnish Technology Development Centre (Helsinki, Finland), Polar Electro Oy (Kempele, Finland), Ab HUR Oy (Kokkola, Finland), the Academy of Finland (Helsinki, Finland) and the Finnish Foundation of Cardiovascular Research.

### Conflict of interest statement

The authors declare that the research was conducted in the absence of any commercial or financial relationships that could be construed as a potential conflict of interest.

## Abbreviations

ΔBP_PostEx_: acute post-exercise change in blood pressure
BMI: body mass index
BP: blood pressure
CAD: coronary artery disease
DBP: diastolic blood pressure
HF: high frequency (0.15–0.4 Hz)
HR: heart rate
LF: low frequency (0.04–0.15 Hz)
MET: metabolic equivalent
SBP: systolic blood pressure
TRIMP: training impulse
VO_2peak_: peak oxygen consumption.
